# Biocontrol Potential of Poly(3-hydroxybutyrate) Fibers Functionalized with Chitooligosaccharide/*Bacillus subtilis* Using Electrospinning and Electrospraying

**DOI:** 10.3390/polym17050692

**Published:** 2025-03-05

**Authors:** Nikoleta Stoyanova, Nasko Nachev, Mladen Naydenov, Iliyana Valcheva, Mariya Spasova, Olya Stoilova

**Affiliations:** 1 Laboratory of Bioactive Polymers, Institute of Polymers, Bulgarian Academy of Sciences, Akad. G. Bonchev St., bl. 103A, 1113 Sofia, Bulgaria; nstoyanova@polymer.bas.bg (N.S.); nachev_n@polymer.bas.bg (N.N.); 2 Department of Microbiology, Agricultural University, 4000 Plovdiv, Bulgaria; mladen@au-plovdiv.bg; 3 Biodinamika Ltd., 4000 Plovdiv, Bulgaria; valchevailiana1@gmail.com

**Keywords:** chitooligosaccharide, poly(3-hydroxybutyrate), *Bacillus subtilis*, electrospinning, electrospraying, biocontrol

## Abstract

Sustainable agriculture increasingly relies on biocontrol agents as eco-friendly solutions to combat plant diseases while improving soil health. In this context, species of the genus *Bacillus*, particularly *Bacillus subtilis*, have shown promise as effective biocontrol agents for plant diseases. This study demonstrates the successful fabrication of biohybrid materials by decorating electrospun poly(3-hydroxybutyrate) (PHB) fibers with electrosprayed chitooligosaccharide (COS) and *Bacillus subtilis* using simultaneous electrospinning and electrospraying. During electrospraying, COS formed a uniform film over the PHB fibers, serving as both an adhesive and a protective coating that maintained the viability and functionality of the embedded bacteria. SEM confirmed that bacterial spores were uniformly spread across the COS-coated biopolymer fibers. ATR-FTIR spectroscopy verified the successful deposition of COS on the fibers, while mechanical assay demonstrated enhancement in mechanical characteristics after the COS film formation on the PHB fibers compared to uncoated PHB. In addition, COS improved the wetting properties of the fibrous PHB material, creating a favorable environment for bacterial growth and development. Microbiological tests showed that the embedded *B. subtilis* remained viable and proliferated normally after 48 h under suitable conditions at 28 °C on agar medium. Furthermore, the biohybrid COS/*B. subtilis*-*on*-PHB materials also effectively inhibited the growth of pathogenic fungi, including species of *Alternaria* and *Fusarium*. These findings highlight the potential of dual electrospinning/electrospraying techniques for the fabrication of eco-friendly biocontrol formulations. The integration of COS coatings with *B. subtilis* provides a promising approach for sustainable agriculture by combining enhanced material properties with effective antifungal activity.

## 1. Introduction

Diseases in cultivated plants are a global issue, causing significant damage to agricultural crops each year [1]. Plant pathogens including bacteria, viruses, and fungi, along with pests, are responsible for destroying over 40% of crops worldwide [2,3]. At the moment, chemical pesticides are widely used to combat these threats [4]. However, their long-term use leads to environmental pollution, poses risks to human and animal health, and contributes to the development of resistance in many pathogens and pests [5,6]. For these reasons, it is crucial to gradually reduce reliance on chemical pesticides in favor of environmentally friendly agricultural formulations. Eco-agriculture promotes a more sustainable approach to plant protection, emphasizing disease and pest prevention through biocontrol methods. This shift requires the development of safe and effective alternatives to conventional pesticides. Therefore, the creation of new ecological solutions, such as the use of living cells to manage diverse plant diseases pests, is really attractive. Advancing these alternatives is a key step toward the widespread adoption of eco-agriculture [7].

Biocontrol, which involves using microorganisms to combat pests and plant illnesses [8], is a potentially effective substitute for synthetic pesticides and essential instrument for comprehensive pest management. Agents of biocontrol, such as viruses, bacteria, yeasts, fungi, and protozoa, naturally occur in the environment and can protect crops directly or indirectly [9]. To ensure effectiveness without harmful side effects, modern plant protection products must integrate their components while adhering to the principle of “*doing no significant harm*”. Beneficial microorganisms like *Trichoderma*, *Penicillium*, *Streptomyces*, and *Bacillus* spp. are effective against pathogenic fungi and insects, leading to increased crop yields and reduced exposure to toxic chemical pesticides [10,11]. A promising approach involves combining these microorganisms with carefully selected, biocompatible, and non-toxic polymers from renewable sources. This innovative strategy could lead to the development of eco-friendly formulations for plant protection, supporting the transition to sustainable agriculture.

Polysaccharides such as cellulose, chitin, chitosan, starch, and pectin are derived from renewable sources and possess biological and functional properties that make them highly attractive for agricultural applications. Moreover, formulations based on natural polysaccharide chitosan can encapsulate and release biocontrol agents, plant growth promoters, bioactive plant compounds, and agrochemicals in a controlled manner, ensuring precise and targeted delivery for enhanced efficiency [12,13,14]. Notably, chitosan with shorter chains demonstrates increased solubility over a broad pH range, making it even more versatile. Chitooligosaccharide (COS), a low-molecular-weight derivative of chitosan (defined by a degree of polymerization ≤ 20) obtained through enzymatic or chemical degradation, exhibits excellent solubility, biocompatibility, and antimicrobial properties [15,16]. COS is particularly effective against foodborne, plant, and animal pathogens, making it valuable in agriculture for disease control and crop protection. Additionally, research is being conducted on chitosan-based natural adhesives and binders, further expanding its applications in sustainable agriculture and environmental protection. The multifunctionality of COS highlights it potential in developing innovative, eco-friendly solutions for modern agricultural challenges.

Recently, in an effort to develop innovative polymeric materials as biocontrol agents for eco-agriculture, we created eco-friendly materials based on electrospun poly(L-lactide) or polyhydroxyalkanoate coated with polysaccharides and embedded with useful microorganisms [17,18,19]. These materials show significant potential for plant protection and growth promotion. Our results demonstrated that COS enhanced the colonization of *T. asperellum* and *B. subtilis* spores. The proposed approach consists of two stages: first, electrospinning of the PHB solution, followed by dip-coating and drying. However, our previous work showed that simultaneous electrospinning and electrospraying might overcome certain restrictions. The resulting fibrous materials exhibited high bactericidal activity against *Escherichia coli* and complete inhibition of *P. chlamydospora* fungal growth [20,21]. Based on these findings, the current study focuses on a one-step original approach that integrates the simultaneous electrospinning of a solution based on PHB and the electrospraying of COS or a COS/*B. subtilis* suspension to create biohybrid fibrous materials. The adhesive and film-forming properties of COS were utilized to attach bacterial cells to the PHB fibers. In addition, the innovative fiber materials’ mechanical, chemical, and biological properties were carefully investigated. Therefore, this study demonstrates that the proposed one-step method and the obtained eco-friendly materials offer an innovative strategy for distributing biocontrol agents in eco-agriculture, reducing the usage of chemical pesticides, and encouraging a more environmentally friendly method of crop protection.

## 2. Materials and Methods

### 2.1. Materials

Poly(3-hydroxybutyrate) (PHB) with an average molecular weight of 330,000 g/mol was provided by Biomer (Schwalbach, Germany). Chitooligosaccharide (COS) with an average molecular weight of 3000–5000 g/mol (Kitto Life Co., Ltd., Pyeongtaeksi, Gyeonggido, Republic of Korea) was used in this study. *N*,*N*-dimethylformamide (DMF), chloroform (CHCl_3_), and potato dextrose agar were supplied by Merck (Darmstadt, Germany). The chemicals used in the present study were of analytical grade. The company Orange Scientific (Braine-l’Alleud, Belgium) supplied the disposable consumables.

Microorganisms *Bacillus subtilis* was acquired from the collection of company Biodinamika Ltd. situated in Plovdiv, Bulgaria. The microorganisms were cultivated in Tryptic Soy Broth (TSB) supplied by Biolife, Milan, Italy at 28 °C on a rotary shaker at 197 rpm for 5 days until full formation of spores. Centrifugation at 6000 rpm and 4 °C for 15 min was used to extract the bacterial spores, which were then twice cleaned with sterile distilled water. The final spore concentration was 1 × 10^10^ spores/mL. Phytopathogenic fungi were cultivated on a standard growth medium (Potato Dextrose Agar) at 28 °C for a week. These fungal cultures were subsequently used to inoculate experiments to assess the inhibitory effects of COS/*B. subtilis*-*on*-PHB samples.

### 2.2. Preparation of Electrospun PHB Fibers Coated with Electrosprayed COS and COS/Bacillus subtilis

Biohybrid mats were obtained using electrospinning and electrospraying at the same time. The resulting materials are denoted with the suffix “-*on*”, indicating that the bioagents are deposited on the fiber surfaces.

For all electrospinning experiments, a 14% (*w*/*v*) solution of poly(3-hydroxybutyrate) (PHB) in a chloroform/*N*,*N*-dimethylformamide (CHCl_3_/DMF = 4:1 *v*/*v*) solvent mixture was used. For electrospraying, an aqueous chitooligosaccharide (COS) solution (0.5 wt.%) was prepared. Additionally, a COS/*Bacillus subtilis* suspension was prepared by mixing 10 mL of bacterial suspension with 10 mL of the COS solution. For comparison, PHB fibers without bioagents were also prepared by electrospinning.

For all electrospinning experiments, a 14% (*w*/*v*) solution of poly(3-hydroxybutyrate) (PHB) in a chloroform/N,N-dimethylformamide (CHCl_3_/DMF = 4:1 *v*/*v*) solvent mixture was used. This solution was made by heating at 60 °C under reflux. The selection of this solvent system was based on achieving the optimal balance of conductivity, surface tension, viscosity, and solvent volatility to ensure the formation of continuous and uniform fibers, as previously reported [20,21]. While chloroform is highly volatile, the addition of DMF helps to moderate the evaporation rate, improve solution conductivity, and maintain viscosity within the desired range. For electrospraying, an aqueous chitooligosaccharide (COS) solution (0.5 wt.%) was prepared. Additionally, a COS/*Bacillus subtilis* suspension was prepared by mixing 10 mL of bacterial suspension with 10 mL of the COS solution. For comparison, PHB fibers without bioagents were also prepared by electrospinning.

Fibrous materials denoted as COS-*on*-PHB and COS/*B. subtilis*-*on*-PHB were produced through electrospinning of the PHB solution and electrospraying of either the COS solution or the COS/*B. subtilis* suspension. Two syringe pumps (NE-300, New Era Pump Systems, Inc., Farmingdale, NY, USA) were used in the setup positioned 180° apart, linked to a common source of high voltage set at 25 kV. Each pump was fitted with a needle of 19 gauge attached to the positively grounded electrode of the high-voltage generator. The PHB spinning solution was delivered at a flow rate of 3 mL/h, with a 25 cm distance from the needle tip to the rotating drum collector. Electrospraying of the COS solution or COS/*B. subtilis* suspension was carried out at a distance of 10 cm between the needle tip and the collector, and a flow rate of 1.5 mL/h. The grounded rotating collector, 45 mm in diameter, was maintained at a speed of 2000 rpm. The electrospinning process was conducted at 25 °C ambient temperature and 49% relative humidity.

### 2.3. Detailed Characterization

In the present study, scanning electron microscopy (SEM) was used to determine the structure of the obtained fibrous mats and bacterial cells. Before being examined with a Jeol JSM-5510 (JEOL Co., Ltd., Tokyo, Japan), all samples were vacuum-coated with gold for 60 s using a Jeol JFC-1200 fine coater. To determine the mean fiber diameter, the standard deviation of the diameter, and the average cell length and width, at least 30 fibers or bacterial cells from the SEM images were quantified using ImageJ software, version 1.54g.

An IRAffinity-1 spectrophotometer (Shimadzu Co., Kyoto, Japan) fitted with a MIRacle™ ATR accessory (diamond crystal) from PIKE Technologies (Fitchburg, WI, USA) was used to perform ATR-FTIR spectroscopic measurements and record the IR spectra of the materials. At a resolution of 4 cm^−1^, infrared absorption spectra were captured in the 600–4000 cm^−1^ range. IRsolution software, version 1.04, was used to adjust all spectra for H_2_O and CO_2_.

Using an Easy Drop DSA20E (Krüss GmbH, Hamburg, Germany), contact angle measurements were carried out at 25 °C to examine the surface wettability of the fiber materials. After applying a sessile drop of deionized water (10 µL) to the surface of fibrous samples (2 cm × 7 cm, cut in the direction of the collector rotation), the average contact angle was determined using a computer analysis. Ten measurements were taken for each sample.

The mechanical properties of the PHB, PHB/COS, and PHB/COS/*B. subtilis* samples were evaluated using an INSTRON 3344 single-column mechanical testing system with Bluehill Universal software, version 3.11, and a 50 N load cell. Tests were conducted at 21 °C and a strain rate of 10 mm/min. The examined specimens measured 400 µm in thickness, 60 mm in length, and 20 mm in width. The linear part of the stress–strain curves was used to calculate the average values of Young’s modulus (E, MPa), tensile strength (σ, MPa), and elongation at break (εB, %), using at least 10 specimens per sample.

### 2.4. Microbiological Studies

The ability of the beneficial bacteria to survive in fibrous materials based on electrospun PHB fibrous mats decorated with COS/*B. subtilis* by electrospraying was evaluated by analyzing their capacity to create colonies on solid TSA medium. Biohybrid samples (disks 16 mm in diameter) were put in Petri plates on top of sterile TSA and cultured at 28 °C. After 48 h, bacterial colonies were found to develop and grow. For comparison, electrospun PHB mats and electrospun PHB with electrosprayed COS were also subjected to microbiological tests.

Dual culture tests were conducted to assess the interaction between the beneficial bacteria embedded in the fibrous disks (16 mm) and pathogenic fungal strains *Fusarium* or *Alternaria*. The tests involved inoculating solid PDA medium in 9 cm Petri dishes with *Fusarium* or *Alternaria*, followed by placing the fibrous disks embedded with *B. subtilis* spores in the center of the dishes. After 7 days of incubation at 28 °C, the colony growth of *B. subtilis* and the fungi (*Fusarium* or *Alternaria*) was observed.

Additionally, the long-term viability after 90 days of storage of COS/*B. subtilis*-*on*-PHB materials was assessed. Before the long-term storage study, the samples were stored in a desiccator at 4 °C. After 90 days, a disk was plated on solid TSA medium and incubated in a thermostat at 28 °C for 7 days.

### 2.5. Analysis of Statistical Data

The results of the present study are displayed as means ± standard deviation (SD). The statistical significance of the data was assessed using one-way analysis of variance (ANOVA) and Bonferroni’s post hoc test in GraphPad Prism software, version 5 (GraphPad Software Inc., San Diego, CA, USA). The following thresholds were used to determine statistical significance: *p* < 0.05 (*), *p* < 0.01 (**), and *p* < 0.001 (***).

## 3. Results and Discussion

The purpose of this study was to develop eco-friendly biohybrid materials as efficient plant disease biocontrol agents using a one-step preparation method. Our previous work demonstrated that the two-step preparation of PHB mats coated with chitosan is an effective method for creating carriers of beneficial bacteria, promoting their growth [19]. However, electrospraying is a relatively simple, affordable, and easily controllable technique enabling the production of extremely thin, defect-free, and more uniform layers compared to those obtained by other methods [22]. Taking advantage of these benefits, we have shown that combining electrospraying with electrospinning is a very promising method for the rapid and efficient one-step fabrication of fibers with nanoparticle-enriched surfaces [20,21,23,24]. To overcome the limitations of the traditional two-step material preparation process, herein, we report for the first time the development of novel biohybrid materials created by electrospraying a suspension containing chitooligosaccharide (COS) and *Bacillus subtilis* and electrospinning a PHB solution simultaneously. This approach enables the creation of PHB fibers with surfaces enriched with beneficial microorganisms. In addition to serving as a natural adhesive that helps bacteria adhere to PHB fibers, COS also provides a supportive environment for the embedded bioagents, ensuring their viability during storage, enhancing their survival in environmental conditions, and supporting their growth when introduced into plant systems or in the presence of moisture. The advantage of the proposed approach lies in the electrospun/electrosprayed PHB/COS matrix, which provides a protective environment for *B. subtilis*, ensuring higher survival rates and gradual bacterial release—unlike free bacterial suspensions that may degrade or wash away. In addition, COS enhances fiber adhesion to plant surfaces or soil particles, enabling sustained biocontrol activity compared to conventional microbial sprays. Furthermore, both PHB and COS are biodegradable, reducing environmental impact compared to synthetic chemical fungicides, making this approach both effective and eco-friendly.

**Figure 1:**
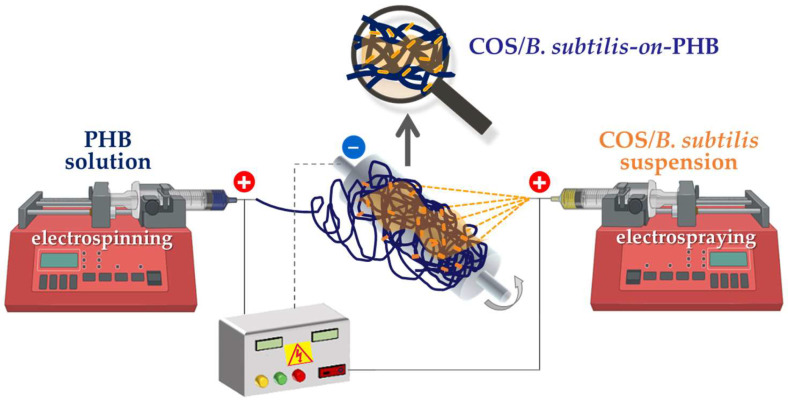
Schematic representation of the simultaneous electrospinning of a PHB solution and the electrospraying of a suspension containing COS and *Bacillus subtilis*.

The aim of the present study was to evaluate the effect of this preparation method on the chemical, mechanical, and biological characteristics of the biohybrid fibrous materials and to assess their biocontrol potential. An illustration of the electrospinning/electrospraying equipment used for the fabrication of these biohybrid materials is shown in Figure 1. As a result, COS-*on*-PHB and COS/*B. subtilis*-*on*-PHB materials were successfully obtained.

### 3.1. Morphology, Chemical Composition, and Wettability

**Figure 2:**
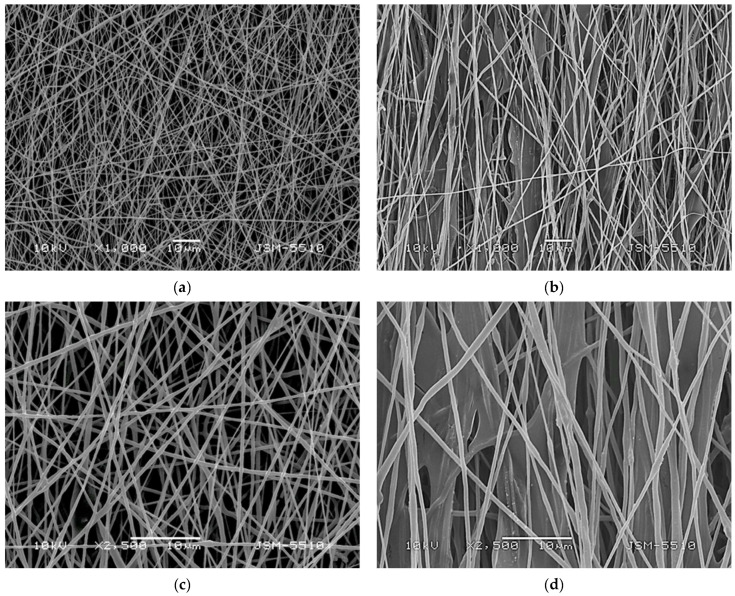
Representative SEM images of (**a**,**c**) electrospun PHB mats and (**b**,**d**) COS-*on*-PHB mats. Magnification ×1000 (**a**,**b**) and ×2500 (**c**,**d**).

SEM was used to examine the surface morphology of the electrospun PHB materials and those made by electrospraying COS solutions and electrospinning of PHB (Figure 2). The results indicated that both the PHB (Figure 2a,c) and COS-*on*-PHB (Figure 2b,d) mats consist of uniform fibers, forming a porous morphology typical of electrospun materials. Apparently, electrospinning of PHB provided the fibrous structure, while electrospraying facilitated COS deposition onto the PHB fibers, along with film formation between the fibers. In support of this, the PHB fibers’ mean diameter was 475 ± 90 nm. In contrast, the PHB fibers decorated with COS had an average diameter of 520 ± 110 nm. This slight enlargement of the fiber’s diameter confirms the deposition of COS onto the PHB fibers, indicating that the formed COS film is extremely thin. As clearly seen, the selected electrospinning conditions, as well as their combination with electrospraying, produced cylindrical fibers with no defects across their length. Moreover, even after electrospraying the COS solution, the desired fibrous structure was preserved.

**Figure 3:**
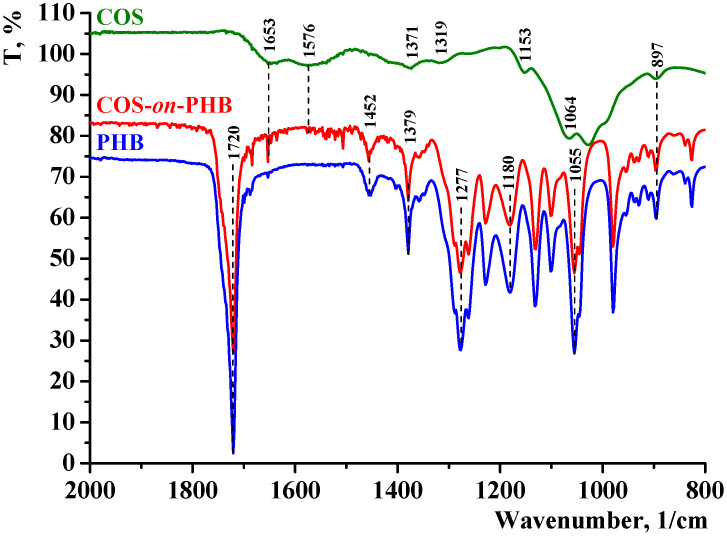
ATR-FTIR spectra of COS (powder), electrospun PHB, and COS-*on*-PHB mat.

The effect of COS electrospraying on the chemical structure of the prepared COS-*on*-PHB materials was evaluated using ATR-FTIR spectroscopy (Figure 3). The representative spectrum of electrospun PHB presents an intense absorption band at 1720 cm^−1^ that corresponds to the stretching vibration of carbonyl groups (C=O), along with a band at 1277 cm^−1^, associated with the bending vibration of the –CH group. Additionally, bands at 1180 cm^−1^ and 1055 cm^−1^ are attributed to the stretching vibrations of the C–O–C groups, while bands at 1379 cm^−1^ and 1452 cm^−1^ correspond to the stretching vibrations of C–H bonds in the –CH_2_ and –CH_3_ groups, respectively. The spectrum of the COS powder (Figure 3) displays characteristic chitosan absorption bands at 1653 cm^−1^ (C=O stretching vibration, amide I), 1576 cm^−1^ (N–H bending vibration, amide II), and 1371 cm^−1^ (bending vibration of O–H and C–N, amide III, from residual N-acetyl groups). Additional characteristic bands are observed at 1153 cm^−1^ and 1064 cm^−1^, which correspond to the stretching vibrations of C–O–C from glucopyranose rings, and a peak at 897 cm^−1^, ascribed to the in-plane bending vibration of the N–H group. In the spectra of electrospun PHB fibers coated with electrosprayed COS (Figure 3), in additional to the characteristic PHB bands, new bands corresponding to the presence of COS were detected. Notably, peaks at 1653 cm^−1^ and 1576 cm^−1^, associated with amide I and amide II, confirm the presence of COS. The low intensity of these peaks is probably due to the very thin COS film deposited onto the electrospun PHB fibers, as confirmed by the average diameter of electrospun PHB fibers with the electrosprayed COS coating. In addition, the characteristic COS peaks around 1000 cm^−1^ overlap with the more intense stretching vibrations of the C–O–C groups in PHB, making them less distinguishable. Furthermore, the bands at 1452 cm^−1^ and 1379 cm^−1^, related to –CH_2_ bending and –CH_3_ symmetrical deformations, are also observed. While ATR-FTIR provides supportive evidence, the confirmation of COS deposition is based on multiple characterization methods, including SEM and mechanical analysis. Nevertheless, the results indicate the presence of COS on the PHB fibers and confirm that the electrospraying process preserved the structural and chemical stability of the prepared COS-*on*-PHB materials.

**Figure 4:**
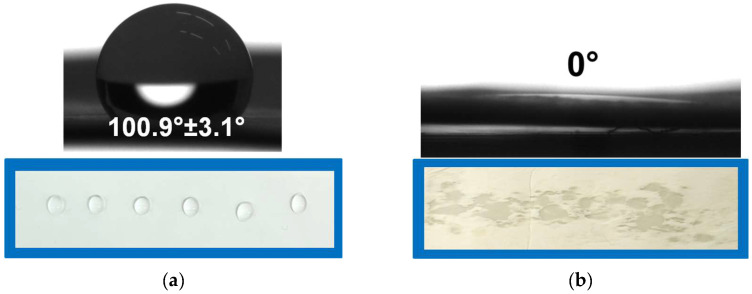
WCA measurements and digital images of the deposited water droplets on the surface of (**a**) electrospun PHB and (**b**) COS-*on*-PHB.

Regarding the possible use of the prepared novel materials as agents for biocontrol, it is essential to design and control their surface properties. An easily wettable surface can provide a favorable environment for embedded bioagents. Therefore, measuring the water contact angle (WCA) and determining the hydrophilic–hydrophobic balance of the material’s surface are critical. Additionally, changes in wettability can serve as further evidence of the successful coating of electrospun PHB fibers through COS electrospraying. The spherical form of the water droplets on the electrospun PHB surface indicated that it had a hydrophobic surface with a water contact angle of around 100° (Figure 4a). In contrast, the electrospinning of PHB solution and electrospraying of chitosan produced a material with a hydrophilic surface (Figure 4b). Water droplets instantly spread across the surface, yielding a contact angle of 0°. This transition from hydrophobic to hydrophilic behavior is attributed to the presence of water-soluble, low-molecular-weight COS uniformly deposited on the PHB fibers. A reduction in the contact angle compared to uncoated electrospun PHB confirms the formation of a hydrophilic COS layer.

### 3.2. Tensile Properties

The obtained results clearly demonstrate that electrospinning provided the fibrous structure, while electrospraying successfully deposited COS onto the PHB fibers and effectively formed films over the electrospun fibers. Therefore, the next step was to investigate the influence of electrosprayed COS into the mechanical behavior of electrospun PHB through tensile testing, as improved mechanical properties are crucial for ensuring the stability and functionality of the material in agricultural applications.

**Figure 5:**
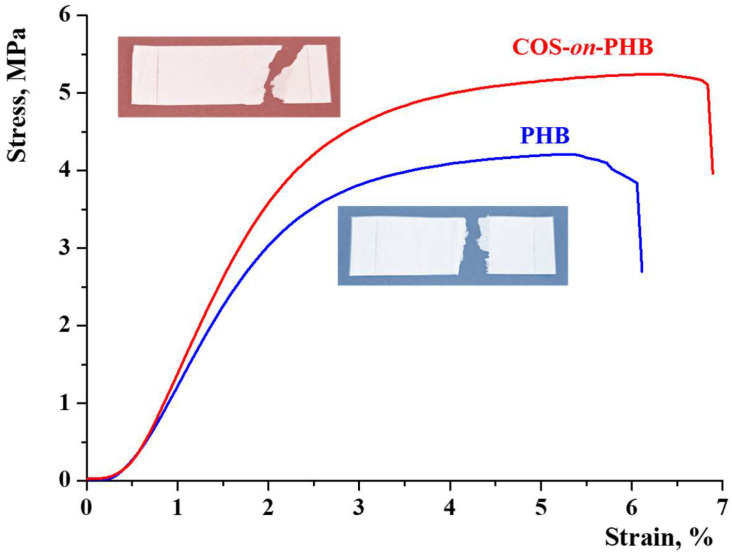
Stress–strain curves of electrospun PHB and COS-*on*-PHB materials during mechanical testing and digital images of the specimens after testing. The specimens were cut in the direction of the collector rotation.

Figure 5 displays the stress–strain curves. Initially, stress is proportional to strain for both samples, followed by an increase beyond the yielding point, reaching maximum tensile stress before tearing. The presence of necking indicates that the fracture behavior is quasi-ductile, while COS enhances the ductility of PHB fibers. It is evident that electrospun PHB fibers coated with electrosprayed COS display better mechanical characteristics than electrospun PHB mat without a coating.

**Figure 6:**
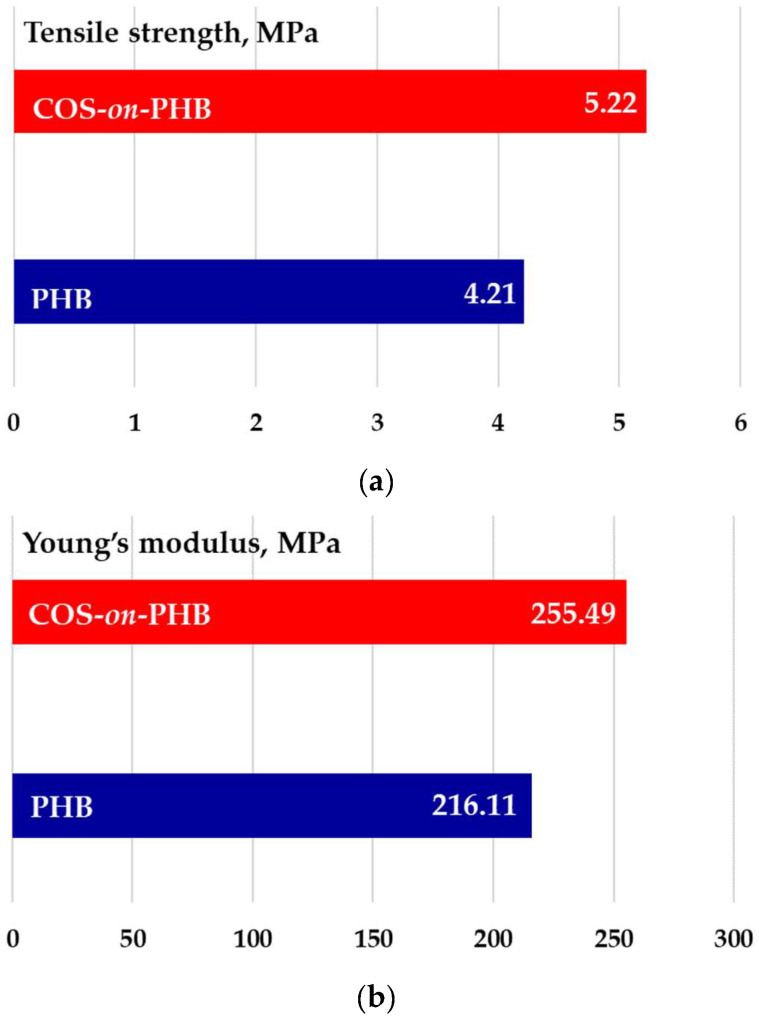
Mechanical characteristics of electrospun PHB and COS-*on*-PHB materials: tensile strength (**a**) and modulus of elasticity (**b**).

This was verified by the average values of Young’s modulus and tensile strength obtained from the stress–strain graphs (Figure 6). As expected, the presence of COS enhances the tensile strength (Figure 6a) and overall mechanical properties of the material by acting as a strengthening agent, forming a thin film and bonding points between the PHB fibers. The electrospun PHB achieved a Young’s modulus of 216.1 MPa and a tensile strength of 4.2 MPa (Figure 6a,b). With COS deposition, these values increased to 5.21 MPa and 255.49 MPa, respectively. These results align with previous studies on chitosan-based coatings in polymer matrices, where similar increases in mechanical performance were reported due to improved fiber bonding and film formation [25]. As seen, the mechanical strength of COS-*on*-PHB is sufficient to maintain structural integrity. While flexibility is essential to prevent cracking, the obtained mechanical values suggest that the material can adhere to plant surfaces and withstand environmental stresses. Furthermore, studies on biopolymeric foliar coatings report mechanical properties within a similar range [19], further supporting the suitability of COS-*on*-PHB formulation.

The obtained results show that the simultaneous electrospraying of COS affects the morphology and wettability of electrospun PHB fibers. Moreover, coating PHB fibers with COS by electrospraying is an easy and efficient technique to improve their mechanical properties. These findings confirm the successful deposition of COS onto PHB fibers and demonstrate that the electrospraying process preserves the structural and chemical stability of the COS-*on*-PHB materials. After confirming the positive effect of electrosprayed COS on the properties of electrospun PHB fibers, the next step in developing biomaterials was the incorporation of beneficial microorganism *B. subtilis* and to assess the biocontrol potential of the prepared COS/*B. subtilis*-*on*-PHB materials.

### 3.3. Morphology of the Prepared PHB Coated with Electrosprayed COS/B. subtilis Mats

**Figure 7:**
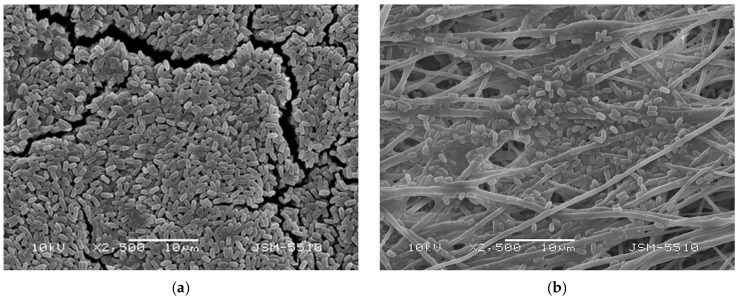
Representative SEM micrographs of (**a**) *B. subtilis* and (**b**) COS/*B. subtilis*-*on*-PHB mats.

The rod-shaped bacterium *Bacillus subtilis* is a model organism for Gram-positive bacteria and has been the subject of extensive study [26,27]. In addition, *B. subtilis* is used as a bioagent as it promotes plant growth; enhances disease resistance; improves soil fertility; and is a naturally occurring, non-toxic microorganism that promotes environmentally friendly agriculture [28,29]. SEM was used to examine the morphology of the *B. subtilis* tested in this investigation (Figure 7a). The rod-shaped spores’ average length and width were found to be 1.484 ± 0.127 µm and 0.736 ± 0.077 µm, respectively.

To prepare biohybrid materials composed of PHB fibers with a surface enriched with *B. subtilis* in a single step, we applied electrospinning of PHB and electrospraying of COS and *B. subtilis* at the same time, as illustrated in Figure 1. The surface morphology of the resulting COS/*B. subtilis*-*on*-PHB materials (Figure 7b) confirms the successful deposition of bacterial spores onto the PHB fibers. Similar to the morphology of COS-*on*-PHB, thin film formation was also observed (Figure 7b). Moreover, due to the presence of COS in the electrosprayed suspension, *B. subtilis* spores were effectively fixed onto the electrospun PHB fibers.

### 3.4. Evaluation of Viability of Embedded Spores in Fibrous Biohybrids

Bacterial viability describes the capacity of bacterial spores to initiate germination and subsequent vegetative growth under defined environmental conditions. It is crucial to assess the viability of *B. subtilis* embedded into electrospun PHB by electrospraying. To evaluate this, uncoated PHB (control), COS-*on*-PHB (control), and COS/*B. subtilis*-*on*-PHB mats were put on agar and cultivated for 48 h at 28 °C. This timeframe was chosen to capture bacterial growth at its peak and observe biofilm formation. After incubation, the Petri dishes containing the samples were photographed, and bacterial growth was assessed.

**Figure 8:**
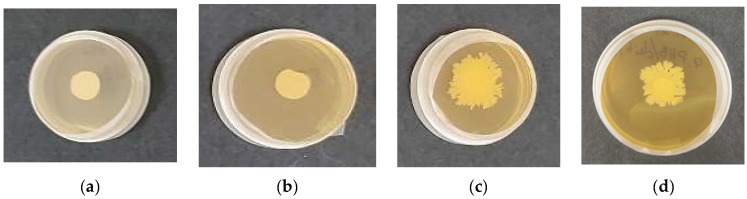
Digital images of (**a**) electrospun PHB, (**b**) COS-*on*-PHB, and (**c**) COS/*B. subtilis*-*on*-PHB after 48 h of incubation and (**d**) the growth of *B. subtilis* after 90 days of storage from COS/*B. subtilis*-*on*-PHB.

As clearly observed, electrospraying the COS/*B. subtilis* suspension does not compromise bacterial viability (Figure 8c). On the contrary, distinguishable *B. subtilis* growth was evident after 48 h of incubation. This confirms that the bacteria remained viable during electrospraying and were capable of normal growth under suitable conditions. Furthermore, the results suggest that the electrospun PHB, when coated with electrosprayed COS, facilitates nutrient diffusion to the bacterial cells, thereby promoting their development. This is probably because COS has a low molecular weight, which enhances its wettability, allowing bacteria to access moisture from the agar more easily and sustain growth. Moreover, this confirms that COS creates a supportive environment for the embedded bioagent, enhancing its survival under environmental conditions, ensuring its viability while being stored, and facilitating its development when moistened. Following this, long-term viability during storage is equally crucial for the practical application of the developed material. To further assess bacterial stability, the viability of *B. subtilis* was tested after 90 days of storage of COS/*B. subtilis*-*on*-PHB mats kept in a desiccator at 4 °C. Following this storage period, the samples were placed in Petri dishes and cultivated at 28 °C for 7 days, after which bacterial growth was evaluated. A digital photograph of the Petri dishes containing the sample is shown in Figure 8d. The results clearly indicate that *B. subtilis* developed normally and spread almost as it did after 48 h, demonstrating that the beneficial bacteria encapsulated by electrospraying onto PHB fibers remained viable, grew, and reproduced even after prolonged storage. These findings highlight the potential of this approach for preserving microorganisms over extended periods. By leveraging the unique properties of COS and PHB, we have developed a novel, inexpensive, and simple method for encapsulating and maintaining viable bacteria in hybrid fibrous materials for at least 90 days.

### 3.5. Two-Strain Culture Analysis

Since the prepared fibrous biohybrid materials were designed to deliver biocontrol agents in eco-agriculture, it is crucial to demonstrate their ability to inhibit the growth of various phytopathogens. It is well known that *B. subtilis* as a beneficial bioagent is capable of combating a wide range of plant pathogens [30]. This capability is multifaceted, involving antibiotic production, competition for nutrients, the synthesis of enzymes that degrade key components of the fungal wall of the cell, and the induction of systemic plant resistance. Furthermore, *Bacillus species* are known to withstand extreme conditions, including variations in temperature, pH, and osmotic pressure. For these reasons, in the present work, a dual-culture test was conducted to evaluate the ability of spores embedded in the electrospun PHB by electrospraying with COS to demonstrate antifungal properties against various phytopathogens. The selected fungal phytopathogens belonged to the genera *Alternaria* and *Fusarium*, both of which are well known as major plant pathogens. In addition to plant damage, these fungi also produce mycotoxins that are harmful and can significantly damage agricultural products [31,32,33].

**Figure 9:**
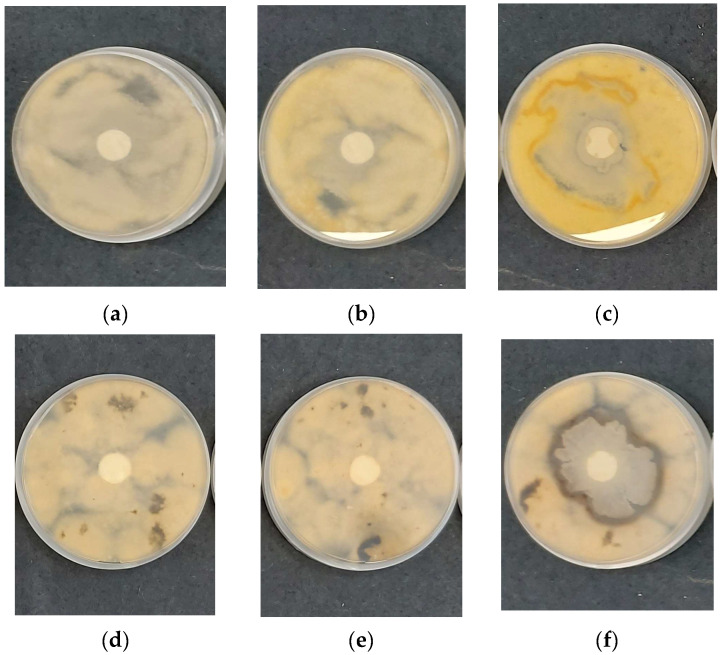
Digital photographs of the discs after 7 days of incubation in the presence of *Fusarium* (**a**–**c**) and *Alternaria* (**d**–**f**): (**a**,**d**) uncoated electrospun PHB mat, (**b**,**e**) COS-*on*-PHB, and (**c**,**f**) COS/*B. subtilis*-*on*-PHB.

In a typical experiment, COS/*B. subtilis*-*on*-PHB samples were initially cut into 16 mm diameter disks and put at the center of Petri dishes that contained a solid PDA medium inoculated with *Fusarium* or *Alternaria*. For comparison, uncoated electrospun PHB and COS-*on*-PHB samples were also tested. The colony growth of the beneficial microorganism *B. subtilis* and the fungal pathogens *Fusarium* or *Alternaria* was measured after 7 days. Figure 9 presents digital images of the electrospun PHB, PHB coated with electrosprayed COS, and PHB fibers coated with electrosprayed COS/*B. subtilis* after incubation in the presence of *Fusarium* or *Alternaria*. Notably, the whole Petri dishes’ surfaces were colonized by fungal pathogens when in contact with the control samples containing electrospun PHB mats. Similarly, significant growth of the fungi was observed when COS-*on*-PHB was tested. In contrast, *B. subtilis* exhibited well-distinguished colony growth around the fibrous discs when COS/*B. subtilis*-*on*-PHB was placed in contact with *Fusarium* or *Alternaria*. Moreover, the presence of *B. subtilis* efficiently suppressed the pathogenic fungal growth. This provides compelling evidence that the developed biohybrid mats, consisting of electrospun PHB fibers coated with electrosprayed COS/*B. subtilis*, possess biocontrol capabilities against *Alternaria* and *Fusarium* species. These findings highlight the potential of dual electrospinning/electrospraying techniques for fabricating eco-friendly biocontrol formulations. The integration of electrosprayed COS coatings with *B. subtilis* offers a promising approach for sustainable agriculture by combining enhanced material properties with effective antifungal activity.

## 4. Conclusions

This study successfully demonstrates the preparation of biohybrid materials by integrating *Bacillus subtilis* and COS onto electrospun PHB fibers using a dual electrospinning/electrospraying approach. The results confirm that COS forms a uniform coating over PHB fibers, serving as both an adhesive and a protective layer that maintains the viability of embedded *B. subtilis*. A SEM analysis revealed a homogeneous distribution of bacterial cells across the COS-coated PHB fibers, while ATR-FTIR spectroscopy proved the successful deposition of COS. Additionally, mechanical studies demonstrated that the COS coating increased the structural integrity of the PHB mats. The incorporation of COS also improved the wetting properties of the fibrous PHB material, fostering a more favorable environment for bacterial survival and proliferation. Microbiological assays confirmed that embedded *B. subtilis* remained viable and capable of normal growth after 48 h and even after 90 days of storage under optimal conditions. Moreover, the biohybrid COS/*B. subtilis*-*on*-PHB materials effectively suppressed the growth of pathogenic fungi, specifically *Alternaria* and *Fusarium*, proving their ability to serve as biocontrol agents. These findings highlight the promise of dual electrospinning/electrospraying techniques for developing sustainable, eco-friendly biocontrol formulations. The integration of COS coatings with *B. subtilis* offers a novel and effective strategy for plant disease management, providing both enhanced material properties and antifungal activity. This approach aligns with the growing demand for novel modern agricultural practices by providing an environmentally friendly solution instead of chemical pesticide usage.

## Data Availability

The data are contained within this article.
